# The effect of a complementary e-learning course on implementation of a quality improvement project regarding care for elderly patients: a stepped wedge trial

**DOI:** 10.1186/1748-5908-7-13

**Published:** 2012-03-02

**Authors:** Lotte Van de Steeg, Maaike Langelaan, Roelie Ijkema, Cordula Wagner

**Affiliations:** 1 NIVEL, Netherlands Institute for Health Services Research, PO Box 1568, 3500, BN Utrecht, The Netherlands; 2 EMGO Institute for Health and Care Research, Free University Medical Centre, PO Box 7057, 1007MB Amsterdam, The Netherlands

## Abstract

**Background:**

Delirium occurs frequently in elderly hospitalised patients and is associated with higher mortality, increased length of hospital stay, functional decline, and admission to long-term care. Healthcare professionals frequently do not recognise delirium, indicating that education can play an important role in improving delirium care for hospitalised elderly. Previous studies have indicated that e-learning can provide an effective way of educating healthcare professionals and improving quality of care, though results are inconsistent.

**Methods and design:**

This stepped wedge cluster randomised trial will assess the effects of a complementary delirium e-learning course on the implementation of quality improvement initiative, which aims to enhance the recognition and management of delirium in elderly patients. The trial will be conducted in 18 Dutch hospitals and last 11 months. Measurements will be taken in all participating wards using monthly record reviews, in order to monitor delivered care. These measurements will include the percentage of elderly patients who were screened for the risk of developing delirium, use of the Delirium Observation Screening scale, use of nursing or medical interventions, and the percentage of elderly patients who were diagnosed with delirium. Data regarding the e-learning course will be gathered as well. These data will include user characteristics, information regarding use of the course, delirium knowledge before and after using the course, and the attitude and intentions of nurses concerning delirium care.

**Setting:**

The study will be conducted in internal medicine and surgical wards of eighteen hospitals that are at the beginning stages of implementing the Frail Elderly Project in the Netherlands.

**Discussion:**

Better recognition of elderly patients at risk for delirium and subsequent care is expected from the introduction of an e-learning course for nurses that is complementary to an existing quality improvement project. This trial has the potential to demonstrate that e-learning can be a vital part of the implementation process, especially for quality improvement projects aimed at complex health issues such as delirium. The study will contribute to a growing body of knowledge concerning e-learning and the effects it can have on knowledge as well as delivered care.

**Trial registration:**

Netherlands Trial Register (NTR): NTR2885

## Background

Given the growing number of elderly within most western countries, the increasing attention that has been paid to this subpopulation is not surprising. Combined with the relatively high use of care associated with elderly patients, this development makes the elderly an increasingly important subpopulation for healthcare organisations. Studies regarding patient safety have shown that elderly hospitalised patients are at greater risk for preventable adverse events than younger patients [1,2], indicating that this growing group of patients will bring specific requirements and risks to hospitals.

In the Netherlands, the publication of a report regarding adverse events in Dutch hospitals [2,3] led to the start of a national patient safety programme in 2008 [4]. The programme consists of several themes or projects, one of which is the Frail Elderly Project. This project is an evidence-based improvement project aimed at improving care for hospitalised elderly (aged 70 and over). Four health problems are addressed, namely, delirium, falls, malnutrition, and physical impairment. In the project, participating hospitals are advised to screen all elderly patients directly after admission for frailty, defined as the risk of developing or the presence of these four health problems (Additional file 1) [5-8]. Hospitals are provided with advice regarding the prevention and treatment of these problems as well. The overall aim of the Frail Elderly Project is to reduce preventable functional decline in elderly patients caused by hospital stay by improving the quality of care. The national programme provides participating hospitals with advice concerning the project through conferences, the programme website, and a guide. The programme does not provide implementation support in the participating hospitals themselves; implementation of the projects lies solely with the hospitals. In our study, we focus on the Frail Elderly Project, with specific attention to delirium.

Delirium occurs frequently in hospitalised patients, especially in the elderly: about 25% of hospitalised patients aged 65 and over experience a delirium during their hospital stay [9]. Depending on the population that has been studied and the methods that have been used, the delirium rate can increase to 62% (surgical patients) or even 87% (intensive care) [10]. Delirium, sometimes referred to as acute confusional state, is a temporary neuropsychiatric disorder with physiological causes. A delirious patient will experience disturbance of consciousness and a change in cognition or disturbance of perception [10]. The disorder is noted for its acute onset and fluctuating state [9], which are key features that separate delirium from dementia. However, given the fluctuating state of delirium and the similarities in symptoms with dementia, depression, and psychosis, delirium is sometimes hard to diagnose [9,11], which creates a problem for providing adequate delirium care. Studies have indicated that between 32% and 67% of delirium cases were missed during hospital stay [12]. Under-detection and subsequent inadequate care present a problem because delirium in elderly patients is associated with increased length of hospital stay, functional decline, admission to long-term care, and higher mortality [13,14].

Nevertheless, several studies have shown that a significant percentage of delirium cases are preventable, and different strategies have reduced by approximately one-third the incidence of delirious patients [15-18]. The Frail Elderly Project aims to decrease the incidence of delirium in hospitalised elderly patients and minimise the adverse effects associated with delirium, such as functional decline. As mentioned earlier, the implementation of the Frail Elderly Project is a task of the hospitals themselves; no onsite support or training is provided by the national programme. Because several studies have shown that both physicians and nurses have a lack of knowledge concerning delirium and its recognition [9,19,20], an educational tool would appear to be a valuable supplement to the delirium part of the Frail Elderly Project. That an educational intervention can significantly improve delirium recognition and care has been shown by a previous study, which resulted in a strong decline in delirium point prevalence -- 9.8% in the intervention group versus 19.5% in the control group [21].

Because knowledge about delirium is relevant for virtually all nurses working within the hospital, an educational approach that can improve knowledge and skills for large groups of people is needed. E-learning is one method available for efficiently educating a large number of people. A review by Cook *et al. *[22] has shown that the use of e-learning or 'internet-based education' compared with no intervention is associated with a positive effect on knowledge, skills, and behaviour of healthcare professionals, as well as on patient outcomes. Less clear-cut results have been found for computer-assisted learning aimed at nurses, suggesting more research is needed in this field [23]. Computer-assisted learning refers to all methods of using computerised technology to facilitate education, including e-learning. Whether e-learning can provide a valuable tool for improving delirium care specifically has yet to be determined, though researchers have suggested e-learning as an important option for improving delirium education [24].

The aim of the study is to assess the effects a complementary e-learning course has on the implementation of the delirium part of the Frail Elderly Project, and thus to see whether the use of e-learning improves the provision of delirium care by nurses. The hypothesis underlying this study is that use of e-learning will increase nurses' knowledge regarding delirium and their willingness to change their behaviour regarding at-risk patients. It is hypothesised that these effects will in turn lead to changes in the care provided: screening of elderly patients for risks, observing at-risk patients for delirium, and taking preventative or curative measures. This protocol describes the methodology used in this study and provides details of the stepped wedge cluster randomised controlled trial design [25].

## Methods

### Intervention

For this study, an e-learning course about delirium will be used that is aimed at nursing staff. This course [26] was developed by a commercial publisher (Noordhoff Publishers), in collaboration with a Dutch hospital. The publisher has been approached by the researchers concerning the use of the e-learning course in this study. Prior to selecting the e-learning for this study, the course was reviewed, while still in development, by the researchers and by the Netherlands Centre of Excellence in Nursing. The content of the e-learning course is consistent with Dutch guidelines regarding delirium care [27,28] and the Frail Elderly Project. The Frail Elderly Project has been implemented by Dutch hospitals independently of this study. The selected e-learning course offers hospitals participating in the study the possibility of supporting their implementation with a course that educates nurses in delirium care (one aspect of the project).

The aims of the e-learning course are on the one hand to create or increase awareness about delirium and the associated risks, and on the other hand to increase knowledge about delirium care. The content of the course consists of information regarding delirium, the risks involved for patients, the recognition of at-risk patients and delirious patients, and the prevention or treatment of delirium. By using fictional patient cases, information is provided in a context familiar to nurses. Throughout the course, users are presented with questions concerning previously given information, providing them with insight into the progress they have made.

During the study, the e-learning course will be offered to nurses in the participating wards online for a period of three months. The estimated time needed per nurse to complete the course, including a knowledge test before and after, is four hours. Nurses can stop the course at any time and continue at a later time. When completing the course and the subsequent knowledge test successfully, nurses will be provided with a certificate stating the completed course and the credits awarded. In cooperation with implementation managers of the publisher, 'kick-off' meetings will be organised in each hospital in order to introduce the e-learning course to the users. The aim of these meetings will be to familiarise users with the way the e-learning works and to encourage them to use and complete the course within the three-month period. After one month and again after two months, users who have not completed the course will receive an e-mail reminder concerning the course to further encourage its use [29]. In addition to these personal reminders, each ward will be provided with a monthly overview of use and completion of the e-learning course.

### Setting

Data will be gathered in 18 Dutch hospitals, all of which are at the beginning stages of the implementation of the Frail Elderly Project at the start of the study (self-reported status). These 18 hospitals include two university hospitals, five teaching hospitals, and eleven general hospitals, varying in size (smallest 138 beds, largest 880 beds) and geographical location. One internal medicine ward and one surgical ward of each hospital will participate in the study.

Nearly all hospitals in the Netherlands were initially invited to participate in the study, excluding specialised hospitals such as eye clinics and cancer hospitals, and hospitals already participating in a separate study related to the Frail Elderly Project. The hospitals that responded most quickly to the invitation were included in the study.

### Study design

The study will be conducted using a stepped wedge cluster randomised trial design. The defining feature of this design is that the intervention will be introduced to all clusters -- or in this instance, hospitals -- in a sequential order [25,30,31]. At the start of the study in May 2011, no hospital will have access to the intervention, while at the end in March 2012 all hospitals will have been given access. This offers an advantage over parallel trials, in which certain hospitals would not receive the intervention. The e-learning course is expected to do more good than harm, making it unethical to withhold the intervention from participating hospitals [25,31].

**Figure 1:**
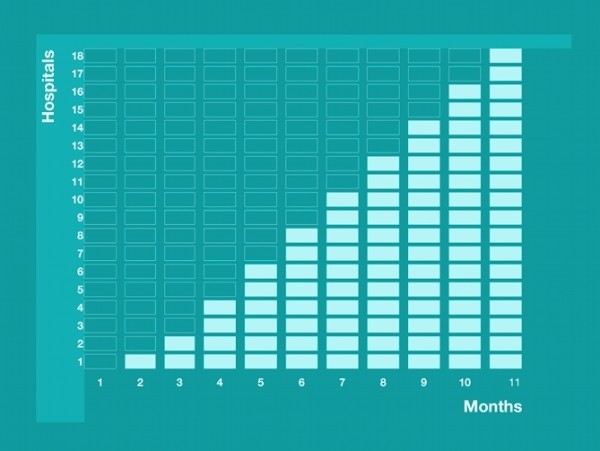
**Diagrammatic illustration of the stepped wedge design**. Each cell represents a moment of data gathering. The dark blue cells represent data gathering in hospitals without e-learning (control period). The light blue cells represent data gathering in hospitals with e-learning (intervention period).

After every monthly data collection moment, one or two new hospitals will receive access to the intervention, as illustrated in Figure 1. The start date at which each hospital is given access to the e-learning course will be randomly assigned, using simple or unrestricted randomisation. Hospitals will each be informed of the start date of the intervention prior to the start of the trial. After a period of 11 months, all 18 hospitals will have received the intervention, and the stepped wedge design will have resulted in gathered data from all hospitals for both the control and the intervention group. This offers an important methodological advantage: each hospital acts as its own control, thereby reducing contamination bias [31].

### Data collection

Monthly patient record reviews of patients 70 years or older will be the main source of data. During 11 months, research nurses will review approximately 10 records per ward per month (20 per hospital), using both nursing and medical records. If a ward provides the research nurse with more than 10 records fitting the inclusion criteria, the nurse will take a random sample from the available records. All record reviews will take place within the same week of the month, using records of patients still in hospital or discharged during the previous week. From these records, data concerning provided delirium care will be gathered, which will offer the following information about the implementation of the delirium aspect of the Frail Elderly Project:

1. Detection of risk for delirium, as well as screening for falls, malnutrition, and physical impairment. The Frail Elderly Project has provided hospitals with screening instruments to assess these risks (Additional file 1). Alternative instruments that are used by hospitals are the Groningen Frailty Indicator [32] and the Identification of Seniors At Risk or ISAR [33].

2. Detection of delirium by nurses using the Delirium Observation Screening scale [34,35] (Table 1).

3. Diagnosis of delirium by physicians.

4. Prevention of delirium by nursing or medical interventions targeting common reversible causes of delirium.

5. Treatment of delirium by starting nursing or medical interventions targeting common reversible causes of delirium or by prescribing medication.

**Table 1:** Delirium Observation Screening scale [34,35]

	The patient:	Never	Sometimes or always
1	Dozes during conversation or activities	0 points	1 point

2	Is easily distracted by stimuli from the environment	0 points	1 point

3	Maintains attention to conversation or action	1 point	0 points

4	Does not finish questions or answers	0 points	1 point

5	Gives answers which do not fit the question	0 points	1 point

6	Reacts slowly to instructions	0 points	1 point

7	Thinks to be somewhere else	0 points	1 point

8	Knows which part of the day it is	1 point	0 points

9	Remembers recent events	1 point	0 points

10	Is picking, disorderly, restless	0 points	1 point

11	Pulls IV tubes, feeding tubes, catheters etc.	0 points	1 point

12	Gets easily or suddenly emotional (frightened, angry, irritated)	0 points	1 point

13	Sees persons/things as somebody/something else	0 points	1 point

For each of three daily shifts the total score is calculated; the total score per shift is a minimum of 0 and a maximum of 13; the total score for a day is a minimum 0 and a maximum of 39. The DOS scale final score is calculated by dividing the total score for the day by 3; the DOS final score is between 0 and 13

The cut-off point is 3; a DOS scale final score of 3 or more indicates a delirium

In addition to data regarding the implementation of the Frail Elderly Project, data regarding the demographic and clinical characteristics of the patients will be gathered.

The record reviews will be carried out by specially trained nurses who have previous experience with reviewing records. An online registration tool will provide these nurses with the means to collect data in the various hospitals. The research nurses will be blinded for the trial condition of the hospitals and are not employed by the hospital where they are conducting the record reviews. Because the e-learning will be used by nurses of the wards participating in the study, the hospitals and wards cannot be blinded to the trial condition.

Besides data from the patient record reviews, data regarding the use of the e-learning course will be gathered. These data include characteristics of the nurses that are given access to the course, such as age, gender, and educational level. Whether the nurses use the course and if so, how often and for how long, will be registered with the web-based course itself. All users will be obligated to complete a delirium knowledge test before and after using the e-learning. These tests consist of randomly selected questions from large question databases. After successfully completing the e-learning and knowledge tests, users will be asked to complete a short evaluation of the course. This evaluation contains five questions regarding the use of the course and the attitude and intention of the user concerning improving delirium care.

### Research population

Records of patients admitted to the participating wards will be included in the study if the patient is 70 years or older and if the length of stay at the time of the record review is at least 24 hours. Only records of patients still in hospital at the time of the record review and patients discharged in the previous week will be included. A total of approximately 3,960 patient records will be reviewed in the course of the study.

### Sampling

The power calculation for the part of the study focused on the effect of e-learning for nurses on provided care (*i.e*., screening for delirium risk) resulted in a power of 0.8, based on the following assumptions: 18 hospitals (36 wards); an improvement of delirium risk screening of 20% (from having 40% of the elderly patients screened to 60%) after the e-learning introduction; an alpha of 0.05; a total of 360 patient records for reviewing per month; an intracluster correlation coefficient (ICC) of 1.0.

For the aspect of the study focused on the effect of e-learning on knowledge, we expect a power of 0.99 assuming the study is conducted in 36 wards (18 hospitals), with an e-learning effect size of 1.00 [22], an alpha of 0.05, at least 30 nurses per ward, and an intraclass correlation of 0.20. When adjusting the effect size to 0.5, a power of at least 0.8 is still achieved.

### Outcomes and data analysis

In the Frail Elderly Project hospitals are advised to screen all elderly patients directly after admission for the risk of developing or the presence of delirium, falls, malnutrition, or physical impairment. The e-learning course educates nurses on screening for delirium. The percentage of elderly patients that are indeed screened for the risk of delirium is the primary outcome of this study. Secondary outcome measures include: the percentage of elderly patients with an increased risk of delirium that are observed with the Delirium Observation Screening scale; the percentage of elderly patients with (an increased risk of) delirium that receive care aimed to prevent or treat delirium; and the percentage of elderly patients that are diagnosed with delirium. Covariates that will be used in the analysis are: age, gender, living situation, comorbidity [36], and admission diagnosis of the patients.

The data analysis will firstly consist of a comparison between hospitals that have received the intervention and hospitals that have not yet received the intervention, according to the stepped wedge schedule. This will be done using multilevel linear regression and multilevel logistic regression analyses. In these analyses, we will adjust for temporal trends and for clustering within hospitals using generalised estimating equations.

Different organisational or system factors that might influence adherence to the intervention or might lead to differences between hospitals in delivered care will be analysed. These factors include hospital type (academic, teaching, general), size, location (urban or rural), and type of records used (paper versus electronic).

### Ethical approval

The Medical Ethics Review Committee of the VU University Medical Centre has approved the study protocol. Patients and representatives on the participating wards will be given the opportunity to object to the use of their (or their relative's) patient record.

## Discussion

The number of studies using a stepped wedge design in order to evaluate the effectiveness of an intervention in routine practice is steadily increasing [25]. The reasons for using the design in this study varied: first, it offered an opportunity to introduce the intervention to all participants, which has both ethical and methodological advantages [25,31]. Second, the fact that the intervention will be made available to all participating hospitals will probably have made participating in the study more attractive to hospitals. Third, by using a stepped wedge design the researchers also aim to avoid a problem experienced in other research regarding changes in healthcare: the fact that the context of the study is constantly changing and that any changes made by an intervention can not be separated from changes due to this dynamic context. The stepped wedge design offers an opportunity to take into account the effect of time on the outcome measures and thus to make a distinction between change that would have occurred without the intervention and change attributable to the intervention [25,37].

This study has several potential limitations. The main limitation of the study is that data on provided care and the behaviour of care providers are gathered from patient records. The risk inherent to this method is that changes found are interpreted as actual changes in care and behaviour, while they might only indicate changes in registration. The e-learning course on delirium that nurses will use in this study does not focus on registration however, but educates nurses in improving the care they provide. Because the screening of patients for increased risks can only benefit patients when all relevant care professionals are aware of the outcomes, screening without proper registration would still imply poor quality of care. In this case, an improvement in registration would also suggest an improvement in care. Still, differences in quality of patient records between hospitals or wards might influence the outcome of this study. Records of poor quality could provide research nurses with insufficient information regarding provided care, which could result in the appearance of poor quality of care.

The presence of the research nurse for the monthly record reviews might influence the professionals working on the wards. In order to minimise bias, hospitals will not be provided with information regarding their performance on the Frail Elderly Project during the research period. Another limitation is that the selection of participating hospitals was based on speed of response to the initial invitation. However, the final group of hospitals provide a fairly complete and balanced overview of hospitals within the Netherlands, varying in type of hospital, size, and geographical location. A final limitation is that hospitals were asked to participate in the study if they considered themselves to be at the beginning stages of implementation of the Frail Elderly Project. Which level of implementation had actually been achieved by the hospitals has not been measured or objectively determined before the start of the study. This indicates that the actual situation in each participating hospital regarding the implementation of the project might vary.

Improving quality of care by implementing evidence-based practice is a difficult process. One way to support this process is by providing healthcare professionals with education regarding the desired change. Education can be a vital part of the implementation process, especially for complex health issues such as delirium. E-learning provides healthcare organisations with a potentially effective and simple way of educating large groups of professionals. This study will contribute to a growing body of knowledge concerning e-learning and the effects it can have on knowledge as well as delivered care.

## Competing interests

The authors declare that they have no competing interests.

## Authors' contributions

CW, ML, and LS collectively developed the study plan. CW conceived of the study, and led the application for current funding through the Dutch Ministry of Health, Welfare and Sport. LS has drafted the initial form and final revision of this manuscript. All authors (CW, ML and RI) have read drafted components of the manuscript, provided input into initial and final refinements of the total manuscript, and agreed to the final manuscript.

## Supplementary Material

Additional file 1 **Screening instruments Frail Elderly Project**. Click here for file
