# Supplementary material for: The effect of a complementary e-learning course on implementation of a quality improvement project regarding care for elderly patients: a stepped wedge trial
Source: Implement Sci. 2012 Mar 2;7:13. doi: 10.1186/1748-5908-7-13 (PMC3310782; doi:10.1186/1748-5908-7-13)
Supplement: Additional file 1 — Screening instruments Frail Elderly Project. [file 1748-5908-7-13-S1.DOC]

**Additional File 1: Screening instruments Frail Elderly Project**

**Delirium**, questions for the patient:

- Do you experience memory problems?
- Have you needed help with self care in the last 24 hours?
- Have you experienced periods of confusion during earlier hospital stay or illness?

One or more questions answered with `yes’ indicate a risk of delirium.

**Falls**, one question for the patient:

- Have you fallen in the past six months?

If this question is answered with `yes’ a risk of falling is indicated.

**Malnutrition**, using the Short Nutritional Assessment Questionnaire to question the patient:

| Did you lose weight unintentionally? |  |
| --- | --- |
| More than 6 kg in the last 6 months | 3 points |
| More than 3 kg in the last months | 2 points |
|  | |
| Did you experience a decreased appetite over the last month? | 1 point |
| Did you use supplemental drinks or tube feeding over the last month? | 1 point |

Two points indicate risk of malnutrition and three points indicate malnutrition.

Or using the Malnutrition Universal Screening Tool:

- Calculate the Body Mass Index of the patient.

BMI Score:

>20 = 0 points

18.5 to 20 = 1 point

<18.5 = 2 points

- Note the percentage of unplanned weight loss in the last 3 to 6 months.

<5 % = 0 points

5% to 10 % = 1 point

>10 % = 2 points

- Establish acute disease effect. If the patient is acutely ill and there has been or is likely to be no nutritional intake for >5 days = 2 points

One point indicates risk of malnutrition, and two points indicate malnutrition.

**Physical impairment**, using the Katz-ADL6 to question the patient:

- Do you need assistance in bathing?
- Do you need assistance getting dressed?
- Do you need help using the toilet?
- Do you use incontinence material?
- Do you need help moving in and out of bed or chair?
- Do you need assistance with eating?

Each question answered with `yes’ indicates dependence and earns one point. A score of two or more indicates risk of functional decline. A score of 6 indicates complete dependence, a score of 4 indicates mild dependence, and a score <2 indicates complete independence.
